# Surgical Outcomes of Vitrectomy with Flower-Petal Fovea-Sparing Inverted Internal Limiting Membrane Flap Technique for Myopic Tractional Maculopathy with Retinal Detachment: A Retrospective Study

**DOI:** 10.3390/jcm14248688

**Published:** 2025-12-08

**Authors:** Hyun Suh, Young-Hoon Park, Young Gun Park

**Affiliations:** 1 Department of Ophthalmology and Visual Science, Seoul St. Mary’s Hospital, College of Medicine, The Catholic University of Korea, Seoul 06591, Republic of Korea; 2 Catholic Institute for Visual Science, College of Medicine, The Catholic University of Korea, Seoul 06591, Republic of Korea

**Keywords:** myopic tractional maculopathy, retinal detachment, internal limiting membrane

## Abstract

**Background/Objectives**: The optimal surgical approach for treating myopic tractional maculopathy (MTM) with retinal detachment remains unclear, particularly owing to complications associated with standard internal limiting membrane (ILM) peeling techniques and macular buckling procedures. Although the flower-petal inverted ILM flap technique is promising for large macular holes, its effectiveness in MTM without macular holes is less understood. We evaluated visual acuity and anatomical recovery in patients who underwent the flower-petal fovea-sparing inverted ILM flap technique for MTM with retinal detachment for 12 months. **Methods**: We retrospectively analyzed clinical data on 22 eyes of 22 consecutive patients diagnosed with MTM involving retinal detachment (Stages 3a, 3b, 4a, and 4b) between May 2019 and May 2023. All patients underwent pars plana vitrectomy using the flower-petal fovea-sparing ILM flap technique. Air, C3F8 gas, or silicone oil tamponade was used. Best-corrected visual acuity (BCVA; logMAR), intraocular pressure, axial length, central retinal thickness (CRT), and foveal contour were assessed using optical coherence tomography preoperatively and at 3, 6, and 12 months postoperatively. **Results**: Mean BCVA (logMAR values) significantly improved (*p* < 0.021). Mean CRT values significantly decreased (*p* < 0.001) at 3, 6, and 12 months. No significant differences in surgical outcomes were observed among tamponade materials. One patient who received air tamponade developed a postoperative macular hole. **Conclusions**: Our findings suggest that the flower-petal fovea-sparing ILM flap technique improves visual function and anatomical outcomes in patients with MTM and retinal detachment. This approach is a promising surgical option for managing MTM with associated retinal detachment.

## 1. Introduction

Myopic tractional maculopathy (MTM) represents a spectrum of pathological changes affecting 9–30% of highly myopic eyes [1]. MTM results from progressive axial elongation of the globe, resulting from continuous stretching and thinning of the sclera. Myopic foveoschisis involves the progressive separation of retinal layers connected by Müller cells and may progress to a macular hole (MH) or retinal detachment. Parolini et al. proposed a new MTM Staging System (MSS) based on nomenclature, pathogenesis, and prognosis, dividing MTM into 12 stages. The staging system supports treatments tailored to the specific stage [2]. Previously, stages 3a, 3b, 4a, and 4b were managed using macular buckling (MB). Pars plana vitrectomy (PPV) with internal limiting membrane (ILM) flap was recommended for stage 1c, such as full-thickness macular hole (FTMH), whereas a combined approach using MB and PPV with ILM flap was suggested for stages 2c, 3c, and 4c.

Recommendations for MB over PPV in stages 3a, 3b, 4a, and 4b were based on the need to reattach the retina to the expanded posterior wall of the globe [3]. MB relieves internal surface vitreous traction and addresses global tractional forces by approximating the posterior scleral wall to the neurosensory retina. This approach reduces the risk of complications previously associated with PPV. MB surgery yields better outcomes than that of PPV in MTM-associated MD. MB aids in reducing both localized vitreous traction and global retinal tension by repositioning the eyeball wall closer to the neurosensory retina. Nonetheless, favorable outcomes have been reported with PPV using ILM techniques in various macular diseases [4,5,6,7]. Furthermore, the limited availability of surgical materials and various situations have restricted the routine use of MB. With ongoing advancements in vitrectomy techniques, PPV is increasingly preferred over MB. Notably, various modified ILM peeling or flap techniques have been introduced to enhance surgical outcomes.

Among these, several modifications of the inverted ILM flap technique reportedly enhance flap stability and retention [8,9,10]. ILM peeling reduces anterior macular traction and eliminates a scaffold for cellular proliferation, potentially minimizing recurrence. However, owing to reduced retinal thickness in highly myopic eyes, postoperative MH formation remains a concern [11]. Fovea-sparing ILM peeling has been proposed to reduce the risk of MH while achieving traction release [12]. Favorable outcomes have also been reported using modified fovea-sparing or ILM flap methods.

The flower-petal inverted ILM flap technique was previously described for large MHs and demonstrated successful outcomes [13]. Multiple small ILM flaps created around the fovea tend to roll toward the fovea to provide coverage without requiring a large central flap as in conventional MH surgery, which simplifies intraoperative management during fluid–air exchange. In eyes with myopic tractional maculopathy, the fovea is often already extremely thin or presents with a lamellar MH. Even after the traction is relieved, the thinned retina within an eye showed its structural support. This significantly increases the risk of progression to a full-thickness MH. Furthermore, this approach would aid in maintaining central ILM to reduce the risk of postoperative MH.

We hypothesized that modified fovea-sparing ILM peeling in cases of myopic foveoschisis without MH could provide favorable anatomical and visual outcomes. Notably, we postulated that this approach could be beneficial in eyes with high myopia and posterior staphyloma without MHs. Herein, we report the long-term outcomes of PPV using the flower-petal fovea-sparing ILM flap technique in patients with MTM-associated retinal detachment in stages 3a, 3b, 4a, and 4b.

## 2. Methods

### 2.1. Study Design and Patients

This study was a retrospective review of consecutive cases. Patients diagnosed with MTM stage 3a, 3b, 4a, or 4b at the Department of Ophthalmology, Seoul St. Mary’s Hospital, Seoul, Korea, between January 2019 and January 2023, and who underwent PPV with the flower-petal fovea-sparing ILM flap technique without MB by a single expert surgeon (Y.G.P), were included. All procedures were conducted in accordance with the Declaration of Helsinki (1964) and its later amendments. The study was approved by the ethics committee of Seoul St. Mary’s Hospital, The Catholic University of Korea (KC25RASI0390). The requirement for informed patient consent was waived due to the retrospective nature of the study. Access for data collection was conducted from 19 December 2024 to 25 March 2025. No personal identification information other than the patient’s medical record number was included during data collection, and all personal identification information was excluded before data processing.

All included patients underwent surgical treatment and were monitored for at least 12 months. Exclusion criteria included a history of ocular trauma or previous ocular surgery (except for cataract operation in the same eye), as well as coexisting ocular diseases such as retinal vein occlusion, diabetic retinopathy, or other retinal diseases.

### 2.2. Clinical Examinations

All patients underwent routine clinical examinations before surgery and during each follow-up. Evaluations included best-corrected visual acuity (BCVA) using the logarithm of the minimum angle of resolution (logMAR), intraocular pressure (IOP) using a non-contact tonometer, and axial length (AL) measured with the IOL Master 700 (Carl Zeiss Meditec AG, Jena, Germany).

Swept-source optical coherence tomography (SS-OCT) scans were acquired using a DRI Triton SS-OCT device (Topcon, Tokyo, Japan) with IMAGEnet 6 software at baseline and during follow-up visits to evaluate the MTM stage and its progression. The 3D Wide protocol (12 × 9 mm^2^) was employed to assess both macular and peripapillary regions, including a horizontal section through the foveal center. The central retinal thickness (CRT) was measured from the ILM to the retinal pigment epithelium in the foveal region. All scans were performed by the same experienced and masked technician. Images with signal strength below 55 were excluded. Two independent investigators (S.H. and Y.G.P.) reviewed all OCT images and determined MTM staging according to the MSS [2]; discrepancies were resolved by consensus.

The demographic and preoperative data, such as age, sex, MTM grading, AL of both eyes, lens status (phakic or pseudophakic), preoperative BCVA, and CRT using OCT imaging to detect the MTM stages, were collected.

### 2.3. Surgical Technique

All surgeries were performed by a single experienced surgeon (Y.G.P). A standard 25-gauge 3-port PPV was conducted using the Constellation Vision System (Alcon Laboratories, Fort Worth, TX, USA) in combination with a wide-angle non-contact viewing system (Resight; Carl Zeiss Meditec AG, Jena, Germany). The ILM was stained using diluted indocyanine green (1.7 mg/mL). Multiple flower-petal–shaped ILM flaps were created using ILM forceps (Grieshaber, Alcon, Fort Worth, TX, USA) starting from the perimacular area (1–2 disc diameters from the fovea) and extending to the foveal center, where the ILM at fovea remained intact.

**Figure 1:**
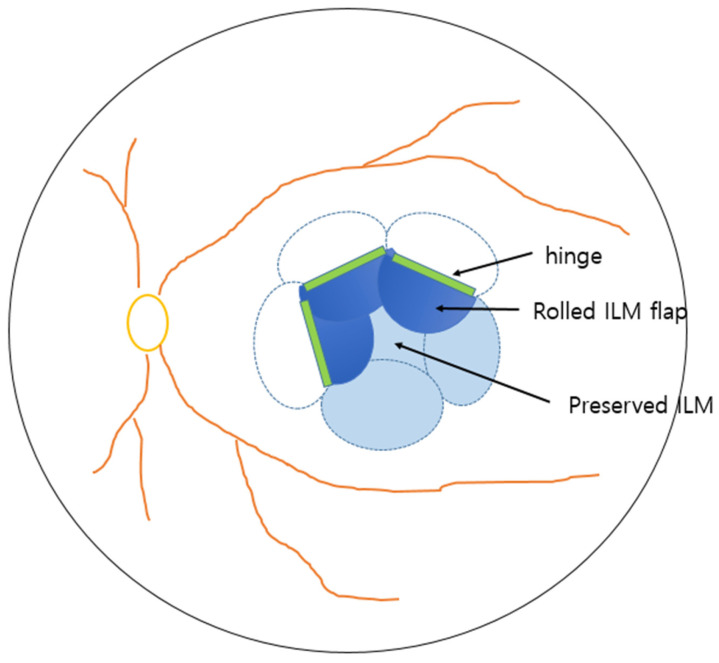
Schematic presentation of flower-petal fovea-sparing internal limiting membrane (ILM) flap technique.

Finally, we used a stream of gentle fluid–air exchange to guide the flaps centripetally toward the fovea. Generally, the use of multiple small flaps enabled natural rolling and approximation toward the hinge region, enhancing foveal coverage (Figure 1). The decision to use air, 14% C3F8 gas, or silicone oil (ARCIOLANE^®^ 5500 centistokes and Oxane, Arcadophta, Toulouse, France) tamponade, and the choice of tamponade agent, were made by the surgeon before and during the operation. Silicone oil tamponade was judiciously selected in limited cases with an increased risk of impending macular hole, poor vision in the fellow eye or inability to maintain prone positioning and where a longer tamponade effect was desired.

### 2.4. Data Analysis

All statistical analyses were performed using SPSS software (ver. 23.0; SPSS Inc., Chicago, IL, USA). For statistical analyses, BCVA values were transformed into the logMAR values. Wilcoxon rank-sum test was used to compare clinical factors based on pre-operative and post-operative results. Spearman’s correlation was used to investigate associations between the tamponade material and visual outcomes. A *p* value of <0.05 was considered statistically significant.

## 3. Results

### 3.1. Baseline Characteristics

Data from 22 eyes of 22 patients, with a mean age of 58 ± 11.28 years, were included in the study. According to the MSS, the demographic and preoperative clinical data of surgical stages was as follows: stage 3a = 7 eyes, stage 3b = 5 eyes, stage 4a = 6 eyes, and stage 4b = 4 eyes (Table 1). At baseline, the lens status of 17 patients was phakic, and that of five patients (22.7%) was pseudophakic. The mean AL of the affected eyes and their fellow eye was 30.5 ± 3.0 mm and 29.9 ± 3.0 mm, respectively. The mean BCVA improved from 1.27 ± 0.41 logMAR at baseline to 0.85 ± 0.31 logMAR at the 12-month follow-up (*p* <  0.05). The mean CRT decreased significantly from 627.2 ± 148.5 μm at baseline to 215.7 ± 79.1 μm at 12 months (*p* < 0.001). C3F8 gas tamponade was used in 15 eyes (68.2%), whereas air and silicone oil tamponades were used in three eyes (13.6%) and four eyes (18.2%), respectively.

### 3.2. Anatomical Outcomes and Visual Prognosis

**Figure 2:**
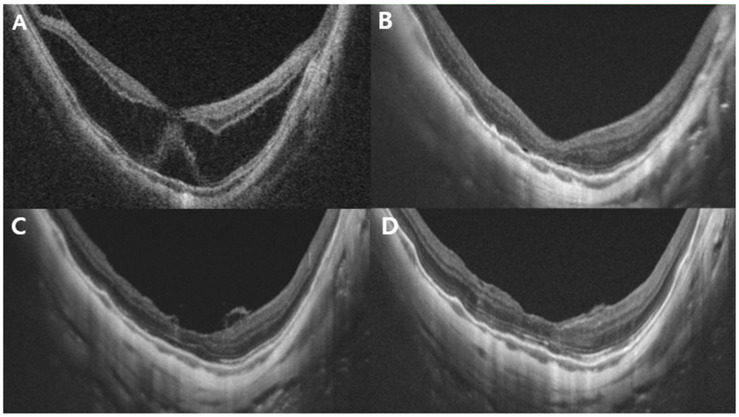
Representative case of a 45-year-old woman with stage 3a MTM. The preoperative AL was 33.32 mm. (**A**) Preoperative OCT image showing MTM with subretinal detachment. The central retinal thickness is 574 μm. (**B**) Postoperative OCT images at 3 months: retinoschisis with subretinal detachment had largely resolved, and CRT was 178 μm. Minimal residual fluid and a blurred ellipsoid zone were observed. (**C**) Six-month postoperative OCT image: the ILM appeared to cover the fovea with flower-petal-like projections of hyper-reflective tissue corresponding to the ILM. (**D**) Twelve months after surgery: the foveal contour remained stable compared with that in the 3-month image, with further recovery of the ellipsoid zone layer. CRT, central retinal thickness; MTM, myopic tractional maculopathy; OCT, optical coherence tomography; ILM, internal limiting membrane.

**Figure 3:**
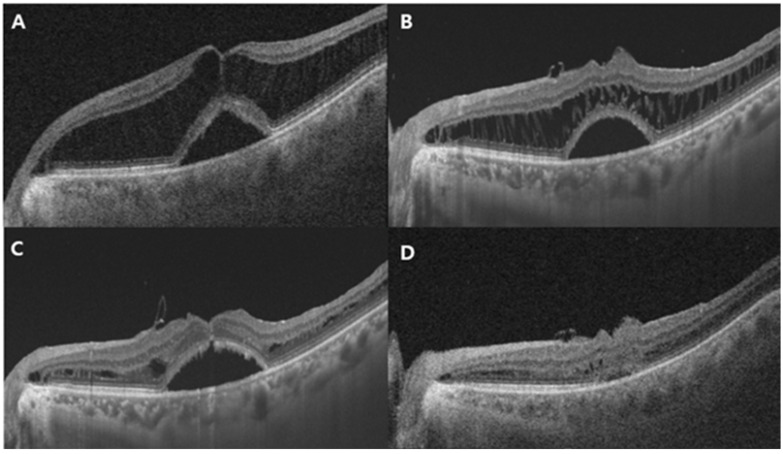
Representative case of a 66-year-old man with MTM stage 3a with air tamponade. (**A**) Preoperative OCT image showing MTM with foveal detachment. The CRT is 638 μm. (**B**) Postoperative OCT image at 3 months: retinoschisis with foveal detachment had slightly improved, but substantial fluid remained. The retinal layers, connected by stretched Müller cells in a multi-stranded configuration, appeared evidently and slightly reduced. (**C**) Postoperative OCT image at 6 months: the multiple stretched linear structures were significantly reduced, and retinal layers showed notable restoration. The ILM was visible covering the fovea; however, foveal detachment persisted. (**D**) Twelve months after surgery: both retinoschisis and remaining foveal detachment had almost completely resolved. CRT, central retinal thickness; OCT, optical coherence tomography.

During the 12-month follow-up period, anatomical macular reattachment was observed in 21 eyes (95.5%). No significant differences in VA or anatomical outcomes were observed among the different tamponade materials (*p* = 0.147 and 0.44). Notably, among the three patients who received air tamponade, one developed an MH with macular detachment and subsequently underwent reoperation with silicone oil tamponade. Another patient in this group required more than 6 months for complete resolution of subretinal fluid (Figure 2 and Figure 3).

Comparison between stage 3 and stage 4 groups revealed a significant difference in both preoperative and postoperative VA (*p* = 0.0147 and 0.025, respectively), whereas no significant difference was observed in CRT (*p* = 0.357 and 0.219). Furthermore, only preoperative VA demonstrated a significant positive correlation with postoperative visual acuity (*p* < 0.001, r = 0.725).

## 4. Discussion

The incidence of myopia has rapidly increased in the Asian population, and pathologic myopia can lead to visual impairment through posterior staphyloma, chorioretinal atrophy, and various stages of MTM [14]. MTM should be considered a spectrum of foveal tractional changes that occur in highly myopic eyes. It typically presents in various forms, such as foveoschisis, retinoschisis, foveal detachment, and lamellar or FTMH, with or without retinal detachment [15]. Parolini et al. organized these different patterns of MTM into the MSS to facilitate understanding and classification of the condition [2].

The anatomical changes in MTM are caused by centrifugal tractional forces with respect to the foveal center. These include both perpendicular and tangential forces. The perpendicular force is induced by scleral elongation as well as anteroposterior vitreous traction, leading to retinoschisis and macular detachment. Conversely, tangential forces result from lateral scleral enlargement and vitreoretinal interface traction, contributing to the development of lamellar holes or FTMH [16].

In 2021, Parolini et al. analyzed data from 157 eyes across different MTM stages and recommended MB as a tailored treatment for stages 3a, 3b, 4a, and 4b while proposing PPV with an ILM flap for stage 1c, and a combination of MB and PPV with an ILM flap for stages 2c, 3c, and 4c [16]. The rationale for MB involved relieving pathological posterior scleral bulging and stretching, whereas PPV primarily reduced tangential traction at the inner retinal surface. MB was shown to be effective, particularly in stages of foveal or macular detachment, without significant epiretinal proliferation or FTMH [17,18].

Nevertheless, PPV remains widely considered to be the preferred surgical approach in MTM; particularly, PPV with various ILM peeling or flap techniques also remains a primary approach in MTM management [19]. Recent advances in various ILM techniques during vitrectomy have enhanced outcomes. ILM peeling removes cortical vitreous, alleviates macular traction, promotes retinal extension, and prevents further surgical complications. However, complete traction release in eyes with high myopia remains challenging owing to their unique anatomical characteristics.

The introduction of the ILM flap technique in 2010 provided a promising option for advanced MTM. The flap functions as a scaffold for glial proliferation and supports tissue bridging in the presence of retinal shortening. Fovea-sparing ILM techniques have shown favorable outcomes over conventional ILM peeling. Meta-analyses comparing fovea-sparing and non-fovea-sparing techniques in myopic foveoschisis indicated significantly better postoperative BCVA and lower rates of postoperative MH formation in fovea-sparing groups compared with that in non-fovea-sparing groups [20,21].

The flower-petal ILM flap technique presents distinct advantages over conventional ILM flap or peeling surgery methods. This procedure enables maximal preservation of the foveal ILM, and no additional intraoperative manipulation is required to secure a large inverted flap, as multiple small flaps naturally roll toward the hinge, forming layered coverage over the foveal region. Additionally, there is no need for measures to keep ILM flaps in place. Although there are limitations inherent to a retrospective study without a comparative control group, we speculate that this configuration might be a helpful in lowering the risk of postoperative MH formation or atrophy. FTMH formation remains a recognized complication following vitrectomy, and recent surgical modifications aim to prevent this. In myopic eyes with thin central fovea, peeling-related trauma may contribute to hole formation. The flower-petal approach facilitates gentle ILM gathering and creates a redundant scaffold, potentially reducing this risk.

In our study, we observed that PPV with the flower-petal ILM flap technique without MB yielded statistically significant improvements in both anatomical and functional outcomes in MTM stages 3a, 3b, 4a, and 4b. Anatomical reattachment was achieved in 95.5% of eyes at the final follow-up. Mean BCVA improved by 0.46 logMAR, from 1.27 ± 0.41 to 0.85 ± 0.31 (*p* < 0.001), and CRT decreased from 627.2 ± 148.5 μm to 215.7 ± 79.1 μm at 12 months postoperatively (*p* < 0.001). This improvement was consistent across stages; no differences were observed based on tamponade material. BCVA did not significantly differ among stages 3a, 3b, 4a, and 4b; however, VA differed significantly between stage 3 and stage 4, likely owing to photoreceptor detachment from the RPE [21,22,23]. These findings suggest that surgical intervention before progression from stage 3 to stage 4 could potentially aid in maintaining visual acuity. Ripa et al. reported secondary operations in 14.5% of eyes due to MB decentration [18]; in our series, only one reoperation was required.

However, our study has some limitations, including a small sample size and retrospective study design. The retrospective design and relatively small sample size limited our ability to perform comprehensive regression analysis to fully control for potential confounding factors. Future prospective studies with larger sample sizes would allow for more robust multivariate analysis. Also, the 12-month follow-up period was relatively short for assessing long-term outcomes. Especially, additional limitation is the lack of detailed information regarding the timing of cataract surgery (simultaneous versus staged procedures). Given that lens status changes can significantly influence postoperative VA, this may have affected our ability to accurately assess visual outcomes attributable solely to the vitrectomy procedure. Also, another limitation is the absence of microperimetry data. Microperimetry would have provided a more comprehensive evaluation of foveal function and retinal sensitivity following surgery with this technique, beyond what visual acuity alone can demonstrate. Future studies incorporating microperimetry would likely provide more clear results regarding postoperative foveal function.

However, our findings provide preliminary evidence supporting the efficacy of the flower-petal fovea-sparing ILM flap vitrectomy in MTM stages 3a, 3b, 4a, and 4b. Future large-scale studies with direct comparisons are warranted to determine which surgical approach offers the optimal balance of efficacy and safety in these stages. Although we observed significant anatomical and visual improvements over 1 year, long-term monitoring is required to evaluate the development of macular atrophy. Additional research is warranted to assess progressive changes in these atrophic regions.

The mechanical force applied during PPV with ILM peeling procedures may still pose a risk to extremely thin foveal tissue in highly myopic eyes [24]. The flower-petal ILM flap technique minimizes mechanical stress on the ILM during peeling, thereby reducing trauma to Müller cells and preserving the foveal glial structure. The technique leaves an ILM flap on the fovea, along with residual epifoveal tissue, further reducing tractional forces and minimizing the risk of inadvertent complete foveal ILM peeling. The multiple petal-like inverted flaps, resembling a cabbage-leaf pattern, may act as a glial scaffold to prevent postoperative MH formation. In contrast to conventional approaches, this method preserves the central fovea using multiple small flaps that roll inward to form a scaffold over the perifovea. Collectively, our findings suggest that PPV with the flower-petal ILM flap technique may represent a viable surgical option for managing MTM stages 3a, 3b, 4a, and 4b.

## 5. Conclusions

The flower-petal fovea-sparing ILM flap technique reported herein effectively improved both visual function and anatomical outcomes in patients with MTM and retinal detachment. Thus, this surgical technique may serve as a promising surgical option for managing MTM with associated retinal detachment.

## Figures and Tables

**Table 1:** Baseline demographic and clinical characteristics of patients with MTM and retinal detachment undergoing PPV with flower-petal fovea-sparing ILM flap technique.

Variable	Overall	Stage 3a	Stage 3b	Stage 4a	Stage 4b
Number of patients	22	7	5	6	4
Mean age (years)	62.3 ± 12.4	65.7 ± 11.4	57.4 ± 15.6	65.8 ± 10.4	57.3 ± 13.7
Axial length (mm)					
Diseased eye	30.5 ± 3.0	28.3 ± 3.6	31.7 ± 2.3	30.7 ± 1.5	32.4 ± 2.7
Fellow eye	29.9 ± 3.0	27.8 ± 3.1	31.4 ± 2.3	29.7 ± 2.4	31.8 ± 2.4
BCVA (logMAR)					
Baseline	1.27 ± 0.41	0.89 ± 0.39	1.54 ± 0.36	1.20 ± 0.55	1.52 ± 0.35
3 months	0.97 ± 0.37	0.76 ± 0.25	1.24 ± 0.43	0.92 ± 0.44	1.08 ± 0.15
6 months	0.84 ± 0.35	0.68 ± 0.22	0.96 ± 0.08	0.82 ± 0.48	1.00 ± 0.28
12 months	0.85 ± 0.31	0.66 ± 0.25	0.96 ± 0.09	0.8 ± 0.5	0.95 ± 0.27
Central macular thickness (μm)					
Baseline	627.2 ± 148.5	695.6 ± 16.42	614.4 ± 44.4	599.5 ± 213.9	523.8 ± 44.9
3 months	261.14 ± 92.34	350.67 ± 107.39	237.00 ± 87.25	224.33 ± 44.71	212.25 ± 35.20
6 months	224.9 ± 93.68	319.00 ± 109.47	205.80 ± 61.87	184.17 ± 63.0	178.25 ± 24.9
12 months	215.7 ± 79.1	271.7 ± 86.8	185.8 ± 66.0	179.0 ± 65.7	176.5 ± 24.2
Preoperative lens status					
Phakia	17	6	4	4	3
Pseudophakia	5	1	1	2	1
Tamponade material					
Air	3	2	1		
C3F8 gas	15	5	3	5	2
Silicone oil	4	□	1	1	2

Data are presented as mean ± standard deviation/standard error of the mean. Abbreviations: BCVA, best-corrected visual acuity; LogMAR, the logarithm of the minimum angle of resolution; □: 0 (zero).

## Data Availability

The data that support the findings of this study are available from the corresponding author upon reasonable request. The data are not publicly available due to privacy or ethical restrictions.

## Abbreviations

The following abbreviations are used in this manuscript:

AL	axial length
BCVA	best-corrected visual acuity
CRT	central retinal thickness
FTMH	full-thickness macular hole
ILM	internal limiting membrane
IOP	intraocular pressure
logMAR	logarithm of the minimum angle of resolution
MB	macular buckling
MH	macular hole
MSS	MTM Staging System
MTM	myopic tractional maculopathy
PPV	pars plana vitrectomy
SS-OCT	swept-source optical coherence tomography
